# Assessment of drug use patterns in terms of the WHO patient-care and facility indicators at four hospitals in Southern Ethiopia: a cross-sectional study

**DOI:** 10.1186/s12913-016-1882-8

**Published:** 2016-11-10

**Authors:** Kassa Daka Gidebo, Temesgen Sidamo Summoro, Zewde Zema Kanche, Eskinder Wolka Woticha

**Affiliations:** 1 School of public health, College of health Sciences and Medicine, Wolaita Sodo University, Wolaita Sodo, Ethiopia; 2 School of Medicine, College of health Sciences and Medicine, Wolaita Sodo University, Wolaita Sodo, Ethiopia

**Keywords:** Drug use patterns, Patient care indicators, Facility indicators, Drugs, Ethiopia

## Abstract

**Background:**

Patient-centered care is now the goal for virtually all healthcare systems. The aim of this research was to evaluate the patient care quality in regard to drug dispensing in four hospitals in southern Ethiopia namely Wolaita Sodo University teaching and referral hospital (WSUTRH), Tercha zonal hospital (TZH), Sodo Christian hospital (SCH) and Dubo St. Mary’s Catholic primary hospital (DSMCPH).

**Methods:**

A cross sectional study was conducted by using the WHO patient care and facility indicators between September 10 and October 20, 2014. Patients who visited the outpatient departments of the four hospitals were selected by systematic random sampling method and interviewed. In total 384 patients were selected based on a rough estimate of proportion of patients visiting to the four hospitals. Facility indicators including the availability of essential drugs list (EDL), national drug formulary, standard treatment guideline (STG) and key drugs were evaluated. Descriptive statistical calculations were performed using SPSS® version 20.0 software.

**Result:**

The mean number of drugs was in the range between 1.9 ± 0.9 to 2.2 ± 2.0. The mean consultation time range was found to be 4.2 ± 1.6 to 4.9 ± 5.0 min whereas the mean dispensing time was ranged from 96.1 ± 52.0 to 152.3 ± 47.6 s. The overall mean number of drug prescribed for the four hospitals was 2.0 ± 1.2 and the mean percentage of medications actually dispensed in the hospitals was thus calculated to be 86.3. The mean percentage of medications clearly labeled was 45.4. Patients who knew their dosage forms accurately were 78.8. Among the four hospitals evaluated only one hospital (25 %) had at least a copy of the Ethiopian essential drug list (EDL), standard treatment guideline for hospitals and drug formulary. The mean availability of key drugs in the hospitals was found to be 65.7 %.

**Conclusion:**

The result of the present study indicates that the patient consulting time, medications labeling and availability of key drugs in the hospitals are inadequate. The medication labeling practice in the four hospitals is unacceptably low. These patient care indicators need a special attention for improvement.

**Electronic supplementary material:**

The online version of this article (doi:10.1186/s12913-016-1882-8) contains supplementary material, which is available to authorized users.

## Background

Health care and quality are inextricably linked, therefore to provide health care services without concern for quality is unprofessional and potentially deadly [1]. Appropriate treatment of commonly occurring diseases and injuries and the provision of essential drugs are the two vital components of the primary health care concept as per the Alma-Ata declaration of 1978 [2].

Drug dispensing is a process that ends with a client leaving a drug outlet with a defined quantity of medication(s) and instructions on how to use drugs. The quantity of drugs dispensed depends on their availability and amount a patient needs for a given condition. Thus information available from dispensers may include: drug(s) prescribed, dose(s) prescribed, mean number of items per prescription, percentage of items prescribed that were actually supplied (an indicator of availability), percentage of drugs adequately labeled, quantity of medications and cost of each item or prescription. These data may be obtained from records kept at the drug outlet either in electronic or manual form [3].

Each of these indicators has its own purpose in drug use evaluation. Patient consulting and dispensing times have the purpose of measuring the time that both medical personnel and dispensing personnel spend with patients in the process of consulting, prescribing and dispensing drugs. The time that prescribers and dispensers spend with each patient may indicate the potential quality of diagnosis and treatment. The percentage of drugs actually dispensed is used to measure the degree to which health facilities are able to provide the drugs which were prescribed, the percentage of drugs adequately labeled help measure whether dispensers included essential information about the medications for patients. Patient knowledge of correct medication helps evaluate the effectiveness of the information given to patients on the dosage schedule of the drugs they receive [4].

The ability to prescribe drugs rationally is influenced by many features of the working environment. Two particularly important components are an adequate supply of essential drugs and access to unbiased information about these drugs. Without these it is difficult for health personnel to function effectively. The world health organization has set criteria used for the evaluation of a health facility. The criteria include availability of essential drugs list or formulary and key drugs [3, 4].

Essential drugs are one of the tools needed to fight ill health. By increasing access to essential drugs and their rational use, we could improve health status and secure development gains [5]. “Essential drugs are those that satisfy the health care needs of the majority of the population; they should therefore be available at all times, in adequate amounts and in the appropriate dosage forms” [6]. This concept was introduced to accelerate the positive impacts of drugs on health status, particularly for developing countries [5, 7]. When investigating drug use, the investigation should determine whether a national essential drugs list, local formulary, or equivalent reference material exists, when this material underwent its most recent revision, and in what form it has been distributed to health facilities [3, 4, 7].

Worldwide, more than half of all medicines are prescribed, dispensed, or sold improperly, and 50 % of patients fail to take them correctly [3–6]. About one third of the world’s population lacks access to essential medicines [8]. A survey conducted in 8 hospitals in southern Ethiopia that investigated their prescription patterns concluded that irrational prescribing, as evidenced by high mean number of drugs prescribed per encounter, high percentage of injections, and high percentage of antibiotic use, was prevalent in the studied region [9]. A more recent study in four hospitals in the same region revealed that there was excessive use of antibiotics and injectable medications in the hospitals [10]. Another study that evaluated the WHO patient care indicators in South West Ethiopia also reported that there was a lower than the set values of patient knowledge on their medications, availability of essential guidelines and key drugs in stock in the health facilities [11].

Assessment of drug use patterns with the WHO drug use indicators is becoming increasingly necessary to promote rational drug use in developing countries [4, 12]. Before activities are started to promote rational drug use, an effort should be made to describe and quantify the situation. Several well-established survey methods are available for this purpose. One such assessment method is a prescribing and patient care survey using the WHO health facility drug use indicators. These quantitative indicators are now widely accepted as a global standard for problem identification and have been used in over 30 developing countries [13].

The aim of this study was to evaluate drug use in terms of WHO patient care and facility indicators in four hospitals located in Southern Ethiopia. The hospitals included were Wolaita Sodo University Teaching Referral hospital (WSUTRH), Tercha Zonal hospital (TZH), Sodo Christian Hospital (SCH) and Dubo St. Mary’s Catholic primary hospital (DSMCPH).

## Methods

A cross sectional study for WHO patient care and facility indicators was conducted on patients who visited the outpatient departments of the four hospitals between September 10 and October 20, 2014.

The sample size calculation was carried out based on the following assumption. The assumptions: Level of confidence 95 %, 5 % margin of error, and P is the proportion of patients who visited the hospitals and took correct patient care. Since there was no previous studies done on drug use in the hospitals, p =50 % was taken to have maximum sample size. Based on these assumptions the actual sample size for the study was computed using the formula for single population proportion:

$$ \mathrm{n} = \frac{{\left(\mathrm{Z}\upalpha /2\right)}^2\mathrm{P}\ \left(1\hbox{-} \mathrm{p}\right)}{{\mathrm{d}}^2} $$

Where, n = sample size, Z α/2 = Critical value = 1.96, P = proportion of patients who had taken correct patient care in the hospitals (0.5) and d = marginal error = 0.05.

Then $$ \mathrm{n} = \frac{(1.96)^2(0.5)\ (0.5)}{(0.05)^2} = 384 $$

In total 384 patients were selected by systematic random sampling in the four hospitals to minimize bias which can be made due to difference in patient load on different days in a week and variation in pattern of hospital visits for chronic care patients. Sampling frame was estimated from outpatient department visits of hospitals in the month prior to data collection.

Therefore, 96 patients from each hospital were selected for observation and interview for patient care evaluation. Consultation and dispensing times were recorded by observation of actual contact with physicians and pharmacists. Number of drugs dispensed against the number of drugs prescribed per encounter was recorded in pharmacy at the point of prescription filling. For medication labeling and patient knowledge about dispensed medicines, packages were observed and patients were interviewed on exiting the pharmacy. Patients who did not receive any medication, below age of 18 and those who were not on ease to respond interview were excluded from this study.

For WHO facility indicators evaluation; information for availability of an essential drug list (EDL), Standard Treatment Guideline (STG), and National Drug Formulary were collected from health professionals in pharmacies, physician diagnosis rooms and in-patient nursing rooms and from the heads of these units. Availability of key drugs was assessed via the stock management software and through observation of the stock in the store.

The data collection was carried out primarily by the outpatient pharmacists and pharmacy technicians in each hospital after appropriate training and orientation. Adequate labeling of dispensed drugs and patient knowledge about the dispensed drugs were assessed by the investigators. Data was collected according to WHO data collection format in order to assess patient care and facility indicators (Additional file 1). The data collection was supervised on a daily basis by the investigators involved in this study. Completeness of the data was checked every day during the data collection period. The data generated for each hospital were entered into a computer using Statistical Package for the Social Sciences (SPSS) version 20.0 software (IBM Corporation, Armonk, NY, USA) to be edited, cleaned, and analyzed. The data were analyzed descriptively and summarized using tables and a bar chart. The findings were compared and contrasted with other national and international studies.

The mean and percentage value of indicator variables were calculated in this study using the following formula:

1. Mean consultation time = sum of all consultation times/total number of patients consulted.
2. Mean dispensing time = sum of all dispensing times/total number of samples.
3. Percentage of drugs actually dispensed = number of drugs actually dispensed/number of drugs prescribed x 100.
4. Percentage of drugs adequately labeled = drugs labeled adequately/drugs prescribed x 100.
5. Percentage of patients aware of the correct dosage = patients with correct knowledge of the dosage of all drugs dispensed/patients interviewed x 100.
6. Percentage of key medicines available in stock = key drugs available in stock/total no. of key drugs x 100.

## Results

All participants responded to the interview. Among 384 participants female participants are slightly higher than male and almost half of the participants are in the age range of 31–64 (Table 1).

**Table 1:** Socio-demographic characteristics of patients who attended the four hospitals in southern Ethiopia, October 20, 2014 (*N* = 384)

Hospitals		TZH N (%)	SCH N (%)	DSMGH N (%)	WSUTRH N (%)	Total N (%)
Sex	Male	47 (48.9)	53 (55.2)	44 (45.8)	37 (38.5)	182 (47.4)
	Female	49 (51.1)	43 (44.8)	52 (54.2)	59 (61.5)	202 (52.6)
Age range	18–30	48 (50.0)	36 (37.5)	39 (40.6)	41 (42.7)	164 (42.7)
	31- 64	45 (46.8)	53 (55.2)	55 (57.3)	44 (45.8)	197 (51.3)
	>64	3 (3.1)	7 (7.3)	2 (2.1)	11 (11.5)	23 (6.0)

### Patient care indicators

The summary of the mean number of drugs prescribed, the mean number of drugs actually dispensed, the mean consultation and dispensing time is presented in Table 2.

**Table 2:** Summary of patient care indicators in four hospitals in southern Ethiopia, October, 2014

Patient care Indicators	Hospitals			
	SCH	DSMCPH	TZH	WSUTRH
Mean number of drugs prescribed ± SD	2.2 ± 2.0	2.0 ± 0.9	1.9 ± 0.9	1.9 ± 0.8
Mean number of drugs actually dispensed ± SD	1.9 ± 2.0	1.7 ± 1.0	1.9 ± 0.9	1.4 ± 0.8
Mean consulting time (in minutes ) ± SD	4.2 ± 1.6	4.9 ± 2.8	4.9 ± 5.0	4.8 ± 2.5
Mean dispensing time (in seconds) ± SD	118 ± 53.8	152.3 ± 47.6	96.1 ± 52	110.4 ± 56

Educational status of patients and their knowledge on the prescribed medications are summarized in Table 3. As it can be seen from the table among the 384 patients interviewed 54 (14.1 %) were illiterate, 175 (45.6 %) had attended or completed primary school education, 85 (22.1 %) had attended or completed secondary school education and 70 (18.2 %) had attended or completed education of a college or above levels.

**Table 3:** Distribution of patients’ knowledge of dispensed drugs and their literacy level in the four hospitals; October, 2014

Knowledge questions on dispensed drugs	Illiterate		Primary school		Secondary School		College and above		Total	
	Yes, N (%)	No, N (%)	Yes, N (%)	No, N (%)	Yes, N (%)	No, N (%)	Yes, N (%)	No, N (%)	Yes, N (%)	No, N (%)
Do you remind the name of drug (s)?	19 (5.0)	35 (9.1)	148 (38.6)	27 (7.0)	75 (19.5)	10 (2.6)	68 (17.7)	2 (0.5)	310 (80.7)	74 (19.3)
Do you know the dose of drug (s)?	15 (3.9)	39 (10.1)	139 (36.2)	36 (9.4)	74 (19.3)	11 (2.9)	70 (18.2)	0 (0.0)	298 (77.6)	86 (22.4)
Do you know the duration of treatment?	11 (2.9)	43 (11.2)	54 (40.1)	21 (5.5)	77 (20.0)	8 (2.1)	70 (18.2)	0 (0.0)	312 (81.3)	72 (18.7)
Do you know the frequency of administration?	19 (5.0)	35 (9.1)	145 (37.8)	30 (7.8)	71 (18.5)	14 (3.6)	67 (17.4)	3 (0.8)	302 (78.4)	82 (21.6)
Do you know the possible side effect (s)?	20 (5.2)	34 (8.9)	153 (39.9)	22 (5.7)	70 (18.2)	15 (3.9)	70 (18.2)	0 (0.0)	313 (81.5)	71 (18.5)

**Fig. 1:**
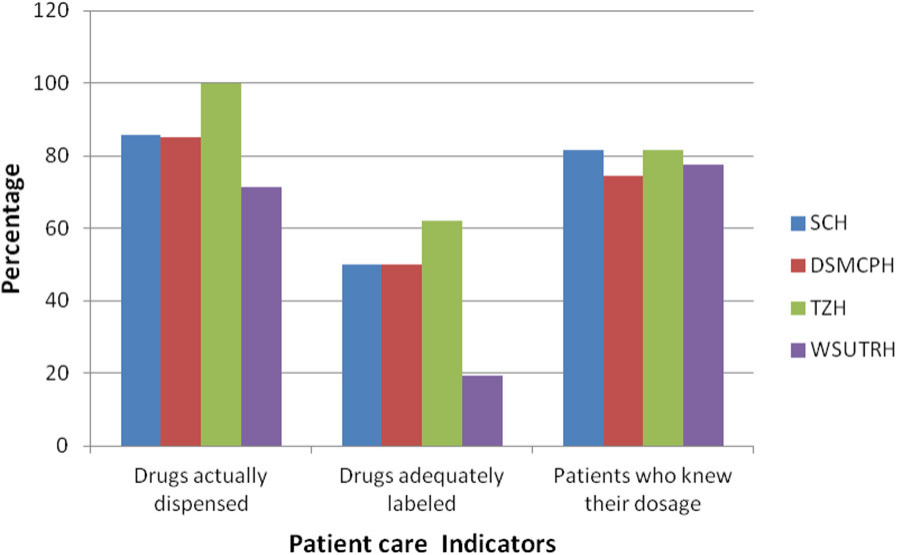
Comparison of some patient care indicators in the four hospitals; October 2014

Percentage of drugs actually dispensed in four hospitals range from 71 % (WSUTRH) to 100 % (TZH), percentage of adequately labeled drugs range from 19.4 % (WSUTRH) to 62 % (TZH) and patients who knew the dosage of the dispensed drugs ranges from 74.5 % (DSMGH) to 81.6 % (TZH/SCH). The percentage of drugs actually dispensed as well as adequately labeled, and patients who knew the dosage of their medication in the four hospitals studied are summarized in Fig. 1 for comparison.

### Facility Indicators (Availability of essential resources)

Except the WSUTRH none of the outpatient pharmacy departments in the hospitals have at least either a copy of National drug formulary, EDL or STG. Availability of the key drugs in the outpatient pharmacy of the hospitals was also evaluated. Percentage of availability of the key drugs for SCH, DSMCPH, TZH and WSUTRH is 62.5, 81.3, 68.8 and 50, respectively (Table 4).

**Table 4:** Availability of key drugs in four hospitals in south Ethiopia, October, 2014

Key Drugs	Hospitals			
	SCH	DSMCPH	TZH	WSUTRH
Amoxicillin	✓	✓	✓	✓
Oral rehydration salt (ORS)	✓	✓	✓	✓
Arthemeter/Lumefantrine	✓	✓	✓	✓
Mebendazole tablet	✓	✓	✓	✓
Tetracycline eye ointment	✓	✓	✓	✓
Paracetamol	✓	✓	✓	✓
Refampicin/Isoniazide/Pyrazinamide/Ethambutol	-	✓	-	-
Medroxyprogestrone (Depo) Injection	-	✓	-	-
Ergometrine maleate injection /tablet	-	✓	✓	-
Ferrous sulphate + Folic acid	✓	✓	✓	✓
Pentavalent.DPT.Hep.Hib vaccine	-	-	✓	-
Adrenaline 0.1 % injection	-	✓	-	-
Antirabies vaccine	-	-	-	-
Oxytocine 10 units	✓	✓	✓	-
Insulin Zinc Suspension	✓	-	✓	✓
Normal saline	✓	✓	-	-
Percentage of availability of key drugs	62.5	81.3	68.8	50

✓ = availability of the item (s); - = absence of the item (s) or stock out

## Discussion

### Consulting and dispensing time

During a consultation, a physician has to make a complete patient evaluation, select the appropriate medications, and enable for proper patient- physicians interaction [14]. The overall mean of consulting time in this study was found to be 4.7 ± 3.0 min (SCH = 4.2 ± 1.6, DSMCPH = 4.9 ± 2.8, TZH = 4.9 ± 5.0, and WSUTRH = 4.8 ± 2.5). This is shorter than what was reported in North West Ethiopia but longer than reported in Jordan and India which were 3.9 and 4 min respectively [11, 14]. Mean consulting time in studies in six European countries was 10.7 min and in the United Arab Emirates it was 10 min [15, 16]. These times are much higher than the results of this present study. Times reported in this current study, as well as in the studies reviewed, are well below the optimal consultation time of ≥ 30 min recommended for conducting proper history-taking, complete physical examination, appropriate health education instructions and prescribing therapy [17]. This current study shows that the consultation time is too short to enable physicians to communicate with their patients regarding their therapy and illness.

Patient compliance directly depends on his/her knowledge about the drug. Therefore an adequate dispensing time is a necessary step towards improving patient care [18]. The mean dispensing time in current study of 119.1 ± 52.4 s is longer than reported in other studies in Ethiopia. It is longer when compared to studies in Jordan, in United Arab Emirates, and in Sharjah which were 28.8 s, 68 s, and 89 s, respectively [11, 16, 19]. A study in Nepal showed 52 s mean dispensing time which is longer than another study in India [20, 21]. The dispensing time observed in these studies is found to be very low compared to the WHO recommendation that a pharmacist should spend at least 3 min in orienting each patient [14, 22]. Since dispensing is terminal step for patient contact with health care provider a pharmacist can hardly explain about the dosage regimen, any side effect of drug therapy and precautions to be taken along with appropriate labeling of envelope in such a short period of time. But a study conducted in Niger reported 195 s which is excellent compared to the WHO recommended dispensing time [14].

### The percentage of drugs actually dispensed

The percentage of drugs actually dispensed is one of the indicators of the availability of essential drugs in health facilities. It may indicate the rationality of drug use in terms of optimum cost. An inadequate drug supply has implications for patients’ health status, is inconvenient for patients and jeopardizes their trust in the health system [18]. The mean percentage of actually dispensed drugs was 86.3 and this is greater than a study report of South West Ethiopia which was 83.4 % [11]. Comparable studies, all conducted in India, reported much lower values than found in our study [14, 23, 24]. We found that the percentage of drugs dispensed in the four hospitals studied, was lower than the standard ideal value (100 %). One study in Saudi Arabia reported 99.6 % of prescribed drugs were dispensed [17].

### The percentage of drugs adequately labeled

Providing adequate information to patients about their drugs is an essential principle of rational pharmacotherapy, since a patient’s level of knowledge about his/her medication is highly associated with a favorable outcome of the therapy. Inadequate labeling may not only result in poor information on drug use but also in poor compliance with the dose regimen [3, 4]. The percentage of drugs adequately labeled ranges from 19.4 to 62.2. The higher value shows that 37.8 % of the medications labeled inadequately whereas 80.6 % were inadequately labeled in the case of the lower value. This shows that there is a very poor medication labeling practice in the hospitals when compared to the standard WHO value which is 100 %. It can be said that an appropriate drug labeling system is not established in any of the hospitals. There was an observed attempt to affix a separate labeling paper on medication containers by some pharmacy professionals in TZH. The WHO recommends that each drug label should include at least the patient name, the name of the drug, the dose regimen and the dose, and the frequency and route of administration [4, 25]. More than a half of the patient medications in this study missed such essential information.

Apart from noting that the medication labeling is poor in this study; the result can be compared and contrasted with other studies. A study in south west Ethiopia revealed that the mean percentage of medications adequately labeled was 70 % which is much better than the result of this study. In a study done by the Federal Ministry of Ethiopia about 43 % the medications were inadequately labeled which is also better than in present study (where in mean 54.6 % of the dispensed medications were inadequately labeled). However, there are similar values which were reported of hospitals in South India and West Bengal in Eastern India [23, 24].

### The Percentage of patients who knew their medications correctly

Dispensing is the end point of contact between pharmacist and patient or the patient's attendant. At this point it is the duty and responsibility of pharmacist to provide adequate information on proper use of drugs [14]. In this study the cumulative mean percentage of patients who knew their medications correctly was 78.8. Table 3 highlights the importance of the literacy level of the patients on their knowledge of the dispensed drugs. As the literacy level increases the knowledge of the patients gets increased.

The above mentioned patient knowledge is lower compared to the WHO recommended value which is 100 %. But it is encouraging in comparison to those values in different local and international studies. For example, in South West Ethiopia the mean percentage of patients who knew their medications in different health facilities was found to be 72.83 [11]. An even lower value was reported (43 %) in a study conducted by Federal Ministry of Ethiopia [26]. A prospective cross-sectional descriptive study in a teaching hospital in Western Nepal, the patient's knowledge on correct drug dosage was reported to be 81 %. Similar studies revealed 52.8 % in Chennai, India, 55 % in Cambodia, 70 % in Brazil and 80.8 % in pediatric patients in India [20]. A recent study in India showed a much lower value of patients’ awareness of their medications [14]. A study in Saudi Arabia revealed 79.3 % of patients had knowledge about their medications [17]. Dispensing practice should mean more than simple issuance of the prescribed or requested items in order to achieve the desired therapeutic goal. The quality and quantity of the dispensed items as well as appropriate drug information determines the success of drug therapy. Pharmacists need to assure patients understand about the medications dispensed for them [27].

### Availability of essential resources

One sign that the concept of essential drugs has been accepted is the development, dissemination and use of a national essential drugs list, a local essential drugs formulary, or equivalent reference material on essential drugs, such as drug information sheets. The availability of such drug information is one cornerstone for rational prescribing [4]. In this study only one out of the four hospitals had a copy of EDL, national drug formulary and STG during the evaluation period. This makes the overall availability 25 % which is much lower than in other studies. A study in south west Ethiopia showed that the availability was 50 % [11]. The availability for 10 health centers in Saudi Arabia was found to be 90 % [17]. However, the availability of the present study can be said to be better than a study in India where 0 % availability was reported [14].

The overall mean percentage of key drugs in stock in the four hospitals was very low (65.7 %) compared with the optimal value (100 %). The minimum to maximum range was between 50 % and 81.3 %. The evaluation was made in terms of the drugs listed in Table 4 most of which are recommended by Federal Ministry of health of Ethiopia as tracer drugs for the hospitals in Ethiopia. The availability of key drugs is lower compared to that of a study reported in India which was 73 %, but greater than that of another study in United Arab Emirates which was 59.2 % [14, 16].

### Limitations

This study examined drug use patterns in terms of the WHO patient care and facility indicators. It only measured the WHO set criteria for patient care to assess the drug use patterns in the hospitals without addressing any of the associated factors affecting the drug use. To determine these factors, further study is needed. Also the actual use of the prescribed drugs by the patients was not included in present study as it is difficult to be sure whether the patients took the drugs or not according to the prescribers’ instruction. The findings of this study only describe the drug prescribing and dispensing patterns in the outpatient departments of the hospitals. Thus it doesn’t reflect the drug use in inpatient wards of the hospitals.

## Conclusion and recommendation

Attention needs to be given to finding a solution to the short consultation times. The consultation time is pivotal in determining appropriate prescribing and use of drugs. Short consultation times, as found in the four hospitals we studied, may negatively impact patient outcomes. Even though the dispensing time in this study was relatively longer compared to other studies still it is shorter than the recommended level. There was very poor labeling of the medications. There is a need of investigating the associated factors with the low patient care parameters. Systemic changes are needed to improve patients’ knowledge of their medications, deliver appropriate patient advice and adequately label of the medications. Essential drug supply management systems should be established to ensure availability of key drugs in the outpatient pharmacies of the hospitals.

## Additional file

Additional file 1:
**A** WHO Patient care Indicators filling format. **B** Check list for partial study of WHO patient care indicators. **C** WHO Facility Indicators filling check list. **D** WHO Facility Indicators filling format. (DOC 463 kb)

## Abbreviations

DSMCPH: Dubo St. Mary’s catholic primary hospital
EDL: Essential drugs list of Ethiopia
FMOH: Federal Ministry of health of Ethiopia
ORS: oral rehydration salt
SCH: Sodo Christian hospital
SPSS: Statistical Package for the Social Sciences
STG: The standard treatment guidelines for hospitals
TZH: Tercha zonal hospital
WHO: World health organization
WSUTRH: Wolaita Sodo University Teaching and referral hospital
