# Supplementary material for: Assessment of drug use patterns in terms of the WHO patient-care and facility indicators at four hospitals in Southern Ethiopia: a cross-sectional study
Source: BMC Health Serv Res. 2016 Nov 10;16:643. doi: 10.1186/s12913-016-1882-8 (PMC5103396; doi:10.1186/s12913-016-1882-8)
Supplement: Additional file 1: — A WHO Patient care Indicators filling format. B Check list for partial study of WHO patient care indicators. C WHO Facility Indicators filling check list. D WHO Facility Indicators filling format. (DOC 463 kb) [file 12913_2016_1882_MOESM1_ESM.doc]

1. **WHO Patient care Indicators filling format**

1. **Check list for partial study of WHO patient care indicators**

Put “X” for none and “√” for yes in blank spaces

1. Number of drugs adequately labeled
2. Name ________
3. Strength _______
4. Dosage form _______
5. Total quantity ______
6. Instructions for the patient on:
   - 1. Frequency of dosing _______
     2. Duration _________
     3. Side effects _________
     4. Precautions _______
7. Date of dispensing _______
8. Expiry date _________
9. Other advisory labels (if any) ______
10. Assessment of patients knowledge of their Medications/dosages
11. Do you remind the name of drug (s)? ___________
12. Do you know the dose of the drug (s)? ___________
13. Do you know the duration of treatment? _________
14. Do you know the frequency of admin.? __________
15. Do you know the possible side effects? ___________
16. **WHO Facility Indicators filling check list**

**Yes No**

1. Availability of copy of EDL _________ ___________
2. Availability of copy of STG __________ ____________
3. Availability of copy of formulary ___________ _____________
4. Availability of key drugs in stock ___________ ____________

Amoxicillin ___________ ____________

Oral rehydration salt ___________ ___________

Artemeter +bLumefantrine __________ _____________

Mebendazole __________ ___________

Tetracycline eye ointment __________ ___________

Paracetamol ___________ ___________

RHZE ___________ ____________

Depo® injection ___________ ___________

Ergometrine maleate injection/tablet __________ ___________

Ferrous sulphate + folic acid __________ ___________

Pentavalent DPT/Hep/Hip vaccine __________ ___________

Adrenaline injection ___________ __________

Ant-rabies vaccine ___________ ___________

Oxytocin 10 units/ml ___________ ____________

Insulin zinc suspension/NPH, 100 units/ml ___________ ____________

Normal saline 0.9% ____________ ___________

1. **WHO Facility Indicators filling format**
